# Organoid Sample Preparation and Extraction for LC-MS Peptidomics

**DOI:** 10.1016/j.xpro.2020.100164

**Published:** 2020-11-25

**Authors:** Emily L. Miedzybrodzka, Rachel E. Foreman, Sam G. Galvin, Pierre Larraufie, Amy L. George, Deborah A. Goldspink, Frank Reimann, Fiona M. Gribble, Richard G. Kay

**Affiliations:** 1 Wellcome Trust – MRC Institute of Metabolic Science Metabolic Research Laboratories, University of Cambridge, Addenbrookes Hospital, Hills Road, Cambridge, UK

**Keywords:** Proteomics, Organoids, Mass Spectrometry

## Abstract

This protocol describes the peptidomic analysis of organoid lysates, FACS-purified cell populations, and 2D culture secretions by liquid chromatography mass spectrometry (LC-MS). Currently, most peptides are quantified by ELISA, limiting the peptides that can be studied. However, an LC-MS-based approach allows more peptides to be monitored. Our group has previously used LC-MS for tissue peptidomics and secretion of enteroendocrine peptides from primary culture. Now, we extend the use to organoid models.

For complete details on the use and execution of this protocol, please refer to Goldspink et al. (2020).

## Highlights

• Preparing organoid cultures, secretions, and FAC-sorted cells for peptidomics
• An established pipeline covering peptide extraction, LC-MS, and data analysis
• Unambiguous detection of closely related peptide hormones
• Semi-quantitative analysis of secreted peptides from stimulated organoid cultures

## Graphical Abstract

**Figure:**
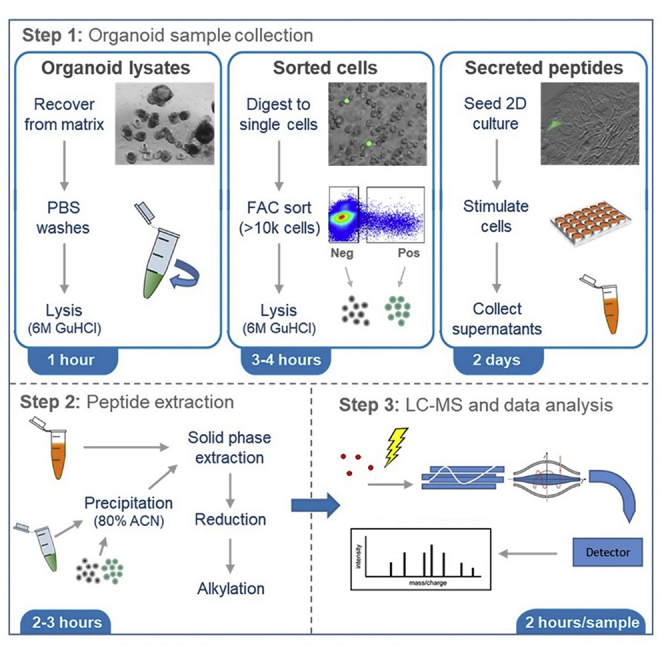

## Before You Begin

### Organoid Culture

**Timing: 2–3 weeks**

This protocol describes the processes and methods involved in the peptidomics analysis of human intestinal organoids, which would be applicable to any organoid line that synthesizes bioactive peptides. However, organoid culture protocols differ significantly depending on the tissue, species, and cell types of interest. Organoids of interest should be generated, cultured, and differentiated using existing protocols. For details of the generation, culture, and genetic modification of human intestinal organoids, please refer to (Fujii et al., 2015).

***Note:*** The techniques we describe here use organoids grown in matrix domes of Basement Membrane Extract (BME) or Matrigel in 6-well tissue culture plates. Depending on the expression level of peptides of interest, one confluent well is typically enough for peptide measurement of organoid lysates or collection of 2D secretion supernatants from two stimulation conditions. The number of organoid wells required for fluorescence-activated cell sorting (FACS) must be empirically determined based on the cell population(s) of interest; we recommend collecting >10,000 cells for each peptidomic analysis.

**CRITICAL:** All organoid culture should be carried out under aseptic conditions in a biosafety hood.

**CRITICAL:** The use of human or animal tissue for research purposes is subject to ethical and legislative restrictions, appropriate approvals must be obtained before commencing work.

## Key Resources Table

**Table:** 

REAGENT or RESOURCE	SOURCE	IDENTIFIER
**Chemicals, Peptides, and Recombinant Proteins**		

Forskolin	Sigma-Aldrich	F6886
IBMX (3-isobutyl-1-methylxanthine)	Sigma-Aldrich	I7018
Guanidine Hydrochloride	Fisher	364791000
Acetonitrile	Sigma-Aldrich	1.00029.2500
LC-MS Grade Water	Fisher	M/0112/17
Formic Acid	Fisher	A117-50
Methanol	Fisher	M/4058/17
Acetic Acid	Fisher	0714500ML
Dithiothreitol (DTT)	Sigma-Aldrich	43819
Iodoacetamide	Sigma-Aldrich	I1149
Ammonium Bicarbonate	Sigma-Aldrich	09830
Bovine serum albumin (BSA; 10% solution in DPBS, low endotoxin, fatty acid free, suitable for tissue culture)	Sigma-Aldrich	A1595
DRAQ5	Invitrogen	15530617
DAPI (4,6-diamidino-2′-phenylindole dihydrochloride)	Sigma-Aldrich	D9542
Advanced DMEM/F12	Invitrogen	12634010
Basement Membrane Extract (BME) Cultrex PathClear Reduced Growth Factor Type 2	Bio-Techne	3533-010-02
Matrigel Basement Membrane Matrix (LDEV-Free)	Scientific Laboratory Supplies	11543550
Glucose	Sigma-Aldrich	G7021
Y-27632 dihydrochloride	Tocris	1254/50
Hank’s Balanced Salt Solution (HBSS)	Sigma-Aldrich	H9394
Fetal Bovine Serum (FBS)	Gibco	10270-106
TrypLE Express	Gibco	12605036
Cell Recovery Solution	Corning	354253

**Deposited Data**		

Sorted cell peptidomics data	PRIDE repository https://www.ebi.ac.uk/pride/	PXD017825

**Experimental Models: Cell Lines**		

hGLU-Venus ileal organoid line	(Goldspink et al., 2020)	N/A

**Software and Algorithms**		

BD FACSChorus	BD Biosciences	N/A
PEAKS (v8.5)	Bioinformatics Solutions Inc	http://www.bioinfor.com/peaks-studio/
Xcalibur (v4.3.73.11)	Thermo Fisher Scientific	https://www.thermofisher.com/order/catalog/product/OPTON-30487

**Other**		

Oasis HLB Prime μElution Solid Phase Extraction (SPE) Plate	Waters	186008052
CellTrics 50 μm disposable filters	Sysmex	04-0042-2317
SPE manifold	Waters	186006961
SPE Dry 96 system	Biotage	SD-9600-DHS-EU
Quanrecovery Extraction Plates	Waters	186009184
UltiMate 3000 nano LC	Thermo Fisher Scientific	ULTIM3000RSLCNANO
Q Exactive Plus Orbitrap mass spectrometer	Thermo Fisher Scientific	IQLAAEGAAPFALGMBDK
Protein LoBind Eppendorf tubes (1.5 mL)	Thermo Fisher Scientific	10708704
Nano LC trap cartridge	Thermo Fisher Scientific	160454
Easy-Spray nano LC column (250 × 0.075 mm)	Thermo Fisher Scientific	ES802A
5 mL Round-Bottom Polypropylene tubes	Corning	352063

## Materials and Equipment

**Table:** Saline Buffer Preparation

Reagent	Final Concentration (mM)	Amount
KCl	4.5	0.3354 g
NaCl	138	8.065 g
NaHCO_3_	4.2	0.3528 g
NaH_2_PO_4_	1.2	0.144 g
CaCl_2_ (1M)	2.6	2.6 mL
MgCl_2_ (1M)	1.2	1.2 mL
HEPES	10	2.383 g
NaOH (1M)	Adjust to pH 7.4	~5 mL
ddH_2_O	n/a	To 1,000 mL
**Total**	**n/a**	**1000 mL**

***Note:*** Saline buffer can be stored at 2°C–8°C for up to 6 months after preparation. 0.001% (w/v) fatty acid-free BSA and glucose (typically 10 mM) are added on day of use.

**Table:** FACS Media

Reagent	Final Concentration	Amount
HBSS	90%	45 mL
FBS	10%	5 mL
Y-27632 (10 mM stock)	10 μM	50 μL
**Total**	**n/a**	**50 mL**

***Note:*** FACS media (without Y-27632) can be stored at 4°C for up to 1 month. Y-27632 should be added on day of use.

**Table:** 6 M Guanidine Hydrochloride

Reagent	Final Concentration (mM)	Amount
Guanidine hydrochloride	6000	5.73 g
LC-MS grade water	n/a	To 10 mL
**Total**	**6000**	**10 mL**

***Note:*** 6 M guanidine hydrochloride can be stored at 22°C for up to 3 months after preparation.

**Table:** 50 mM Ammonium Bicarbonate

Reagent	Final Concentration (mM)	Amount
Ammonium bicarbonate	50	1.97 g
LC-MS grade water	n/a	500 mL
**Total**	**50**	**500 mL**

***Note:*** 50 mM ammonium bicarbonate (aq) can be stored at 22°C for up to 3 months after preparation.

**Table:** 10 mM DTT in 50 mM Ammonium Bicarbonate

Reagent	Final Concentration (mM)	Amount
DTT	10	0.0154 g
Ammonium bicarbonate (50 mM)	50	10 mL
**Total**	**n/a**	**10 mL**

***Note:*** This solution is prepared fresh immediately before use.

**Table:** 100 mM Iodoacetamide in 50 mM Ammonium Bicarbonate

Reagent	Final Concentration (mM)	Amount
Iodoacetamide	100	0.1849 g
Ammonium bicarbonate (50 mM)	50	10 mL
**Total**	**n/a**	**10 mL**

***Note:*** This solution is prepared fresh immediately before use.

### LC-MS System Setup

• Samples are loaded onto a 0.3 × 5 mm peptide trap column (Thermo Fisher Scientific) at a flow rate of 30 μL/min and analyzed using a 250 × 0.075 mm Easy-Spray column (Thermo Fisher Scientific). Both nano and trap column temperatures are set at 45°C during the analysis.
• The mobile phases are A: 0.1% formic acid in water (v/v) and B: 0.1% formic acid (v/v) in 80:20 acetonitrile:water (LC-MS grade). Chromatographic conditions are initially 2.5% B, held for 15 min, followed by an increase to 50% B over 90 min, the column is then washed with 90% B for 20 min before returning to starting conditions for a further 20 min, totaling an entire run time of 130 min at a flow rate of 300 nL/min (Table 1).

**Table 1:** Liquid Chromatography Gradient Parameters

Retention (min)	Gradient (% B)	Flow (nL/min)
0.00	2.5	300
15.00	2.5	300
17.00	7.5	300
90.00	50.0	300
91.00	90.0	300
109.00	90.0	300
110.00	2.5	300
130.00	2.5	300

**CRITICAL:** Safety precautions must be taken, and appropriate PPE (i.e., laboratory coat, goggles and gloves) worn when preparing LC solvents. Acetonitrile is harmful if in contact with skin. Formic acid is highly corrosive to eyes, skin, and the respiratory system and should therefore always be added in a fume hood.

• Eluted peptides are directly introduced into the mass spectrometer by positive nano electro-spray ionization using a spray voltage of 1.8 kV, and an S-lens setting of 70 V.
• The mass spectrometer is set to perform full MS scans at 1 microscan with a resolution of 70,000, over a mass range of 400–1,600 *m/z*. Following each survey scan, the top 10 ions of each spectrum are selected for MS/MS analysis, and ions selected for fragmentation are added to an exclusion list for 60 s (Table 2).

**Table 2:** LC-MS/MS Parameters

Parameter	Value
Runtime	0–130 min
Polarity	positive
Default charge state	4
Mass analyzer	Orbitrap
Orbitrap resolution	70,000
Scan range (*m/z*)	400–1,600
Automatic Gain Control (AGC) target	3e6
Micro scans	1
Number of dependent scans	10 (from most intense precursors)
Resolution	17,500
Maximum fill time	300 ms
Mass range	fixed lower *m/z* = 100
AGC target	1e5
Microscans	1
Normalized collision energy (%)	24, 28
Charge exclusion	exclude unassigned and +1 charge state
Dynamic exclusion	60 s
• Data are acquired with Xcalibur software version v4.3.73.11.

***Alternatives:*** This protocol is illustrative of our specific LC-MS/MS approach using a Thermo Fisher Scientific Ultimate 3000 nano LC system coupled to a Q Exactive Plus Orbitrap mass spectrometer. However, alternative LC-MS systems with similar capabilities (nanoflow, mass accuracy, resolving power and *m/z* range) should be suitable.

**CRITICAL:** The instrument should be regularly calibrated, and performance validated with frequent in-house quality controls prior to any sample analysis. See Troubleshooting 1.

## Step-By-Step Method Details

### Recovery and Lysis of Organoids

**Timing: 1 h**

These steps describe the recovery of organoids from matrix domes and subsequent lysis in 6 M guanidine hydrochloride.

1. Recover organoids from matrix domes.
  a. Aspirate organoid culture media.
  b. Add 2 mL of ice-cold Cell Recovery Solution (Corning) per 6-well.
  c. Use a P1000 micropipette to scrape and collect organoids into LoBind 1.5 mL Eppendorf tubes.
  d. Incubate on ice for 30–60 min, inverting halfway through to mix organoids, to allow matrix to dissolve.
  e. Centrifuge at 600 × *g* for 5 min to pellet organoids.
  f. Discard supernatant (including any matrix on top of the pellet).
2. Wash organoids with ice-cold PBS.
  a. Add 1 mL ice-cold PBS to each tube.
  b. Gently rotate and invert tube to resuspend organoid pellet.
  c. Centrifuge at 600 × *g* for 5 min to pellet organoids.
  d. Discard supernatant (including any matrix on top of the pellet).
  e. Repeat steps a-d until no visible matrix remains.

***Note:*** Using ice-cold Cell Recovery Solution and PBS aids with the removal of organoids from extracellular matrix (of the BME domes) and also reduces peptide degradation.

**CRITICAL:** Avoid touching organoids while removing supernatant as they will readily stick to pipette tips. BSA should not be added above 0.001% to prevent sticking as this will interfere with the LC-MS analysis.

**CRITICAL:** Remaining matrix may interfere with peptide extraction and LC-MS. Organoids should be washed with ice-cold PBS until no visible matrix remains (normally 2–3 times).

3. Add 25 μL of 6 M guanidine hydrochloride per organoid dome to resuspend pellet (typically 300–500 μL is used per confluent 6-well) and snap freeze on dry ice.

**Pause Point:** Samples can be frozen at −70°C until ready to continue with peptide extraction

### Peptide Extraction of Lysed Organoids

**Timing: 21 h**

This method describes peptide extraction of whole lysed organoid cultures.

4. Defrost the samples on ice and precipitate with a 1:5 ratio of 80% acetonitrile (ACN) in water.
  a. If necessary, transfer samples to a larger Eppendorf tube prior to precipitation.

***Note:*** Precipitation will result in the formation of three layers within the sample; organic, aqueous, and solid cell precipitate. Centrifugation and placing the sample on ice will clarify the layers, see step 5.

5. Centrifuge at 3,500 × *g* for 5 min at 4°C and transfer the aqueous phase (lower), without disturbing the precipitate, to a clean LoBind Eppendorf tube.
6. Evaporate the supernatant in a rotary evaporator until dry (usually takes ~18 h; overnight evaporation).
7. Reconstitute in 250 μL 0.1% formic acid in water (v/v) and transfer onto an Oasis HLB Prime μElution solid phase extraction (SPE) plate (Waters) and gently apply positive pressure on an SPE manifold.
8. Wash all samples with the following reagents, ensuring the SPE plate reservoirs are clear before each step.
  a. 200 μL 0.1% formic acid in water (v/v).
  b. 200 μL 5% methanol:1% acetic acid in water (v/v).
9. Elute the samples into a clean QuanRecovery plate with two 30 μL aliquots of 60% methanol:10% acetic acid in water (v/v).
10. Evaporate the supernatant, under nitrogen at 40°C on a Biotage SPE Dry 96 system until dry.
11. Reconstitute into 75 μL 10 mM DTT in 50 mM ammonium bicarbonate.
12. Incubate the samples for 1 h at 60°C.
13. Add 20 μL 100 mM iodoacetamide in 50 mM ammonium bicarbonate.
14. Incubate for 30 min at 18°C–25°C, in the dark.
15. Dilute the samples with 25 μL 1% formic acid in water (v/v).

***Note:*** Reduction and alkylation (steps 11–14) breaks disulfide-bonded peptides so they can be identified in the bioinformatics analysis. These steps can be removed but this will reduce the number of positive peptide matches. If a targeted quantitation approach is being used, the SPE extract is to be diluted with 75 μL 0.1 % formic acid in water (v/v) after step 9 for LC-MS/MS analysis.

### Purification of Cell Populations by FACS

**Timing: 3–4 h**

This method describes the isolation of cell populations by FACS based on the presence of fluorescent reporters.

16. Recover organoids from matrix domes.
  a. Aspirate organoid culture media.
  b. Add 2 mL of ice-cold Advanced DMEM/F12 (ADF) per 6-well.
  c. Use a P1000 micropipette to scrape and collect organoids into 15 mL centrifuge tube(s).
  d. Centrifuge at 400 × *g* for 5 min to pellet organoids.
  e. Discard supernatant.
17. Digest organoids to single cells.
  a. Resuspend pellet in 5 mL of TrypLE Express per 6-well.
  b. Incubate in a water bath at 37⁰C for 20 min.
  c. Mechanically triturate organoids 10–20 times each with P1000 and P200 micropipettes.
  d. Take a small drop (~20 μL) of the cell suspension onto a Petri dish and check under a microscope for the presence of single cells.
  e. Repeat steps b-d, checking digestion at 5–20 min intervals, until a largely single-cell suspension is obtained.

***Note:*** Digestion to single cells typically requires 30–60 min incubation with TrypLE at 37⁰C, depending on organoid size and morphology. To enhance digestion, organoids can be transferred to a 50 mL tube during step 17 (which prevents organoids settling to the bottom of the tube) but should be returned to 15 mL tubes for centrifugation. Some small clusters of up to ten cells are acceptable at this stage as they will be further broken up by mechanical trituration.

18. Wash organoids with FACS media.
  a. Centrifuge at 400 × *g* for 5 min to pellet single cells.
  b. Discard supernatant.
  c. Mechanically triturate organoids 10–20 times each with P200 and P20 micropipettes.
  d. Resuspend pellet in 5 mL of FACS media.
  e. Repeat steps a-d two more times, then pass cell suspension though a 50 μm cell strainer.
19. Repeat steps 18a–c and resuspend in FACS media supplemented with live cell dyes (for example, 5 μM DRAQ5 and/or 2 μg/mL DAPI).
20. Transfer single-cell suspension to 5 mL round bottomed polypropylene FACS tubes.
21. Sort cell populations of interest directly into 350 μL of 6 M guanidine hydrochloride in LoBind 1.5 mL Eppendorf tubes.

**Figure 1:**
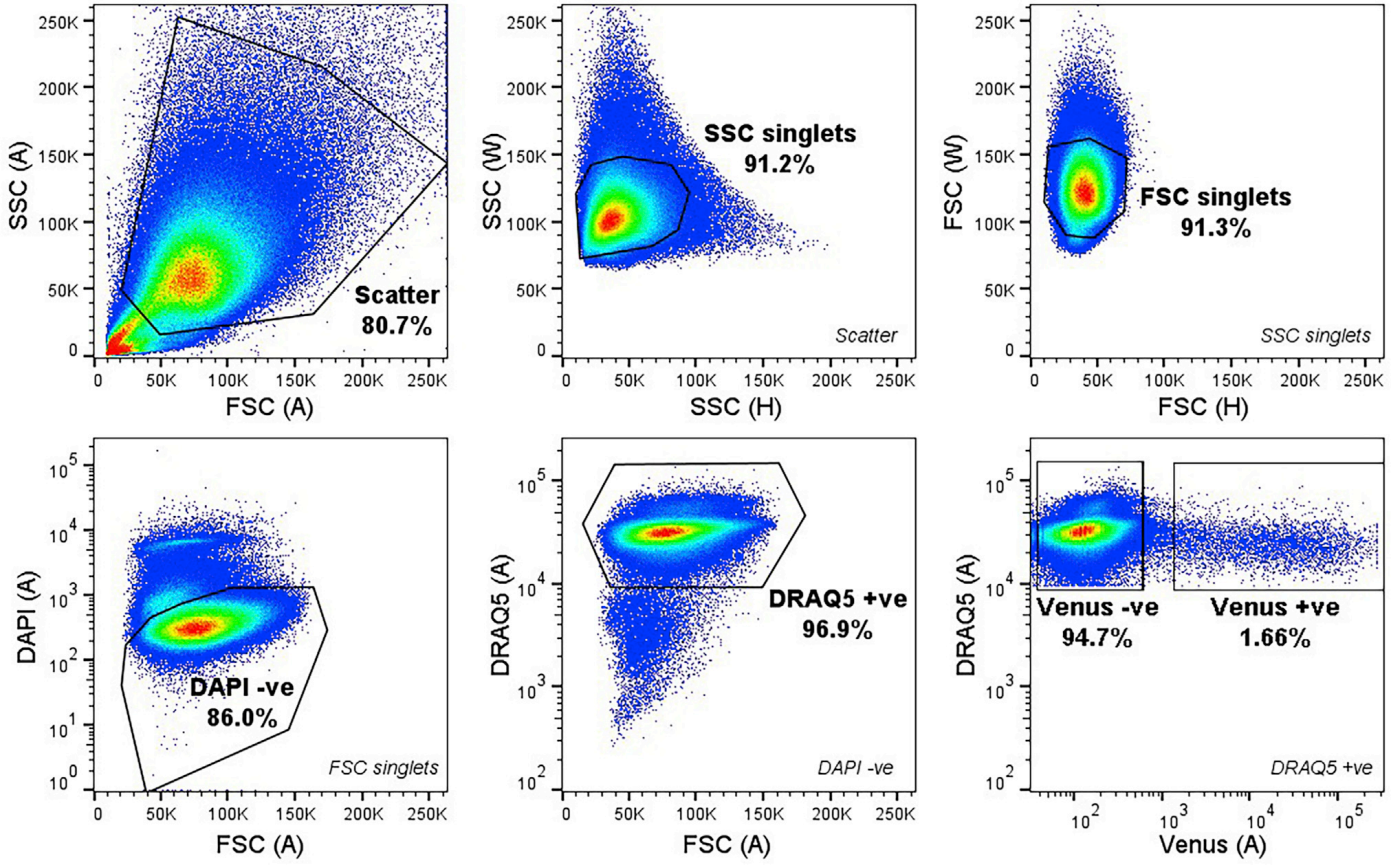
Successful and Unsuccessful 2D Organoid Cultures 2D organoid-derived cultures observed following overnight incubation and thorough washing with saline buffer. Scale bar, 200 μm. Successful cultures can be high (A) or low (B) density. Unsuccessful cultures can adhere as single cells (C) or fail to flatten (D).

***Note:*** Where possible we recommend collecting >10,000 cells per population for peptidomic analysis, although we have previously measured high-abundance peptides from as few as 100 cells from fresh tissue. Follow manufacturer instructions regarding use of cell sorters. See Figure 1 for an example FACS sort layout.

22. Vortex briefly and then immediately snap freeze cells on dry ice after sorting.

**Pause Point:** Samples can be frozen at −70°C until ready to continue with peptide extraction

***Note:*** As there is 10% FBS present in the FACS media, some bovine derived peptides will be carried through to LC-MS analysis. The impact on peptidomics is minimal, however for proteomic analysis the bovine peptides will generate interferences to the database search. If interested in proteomics analysis, use 0.1% BSA or a lower concentration of FBS in FACS media.

**Figure 2:**
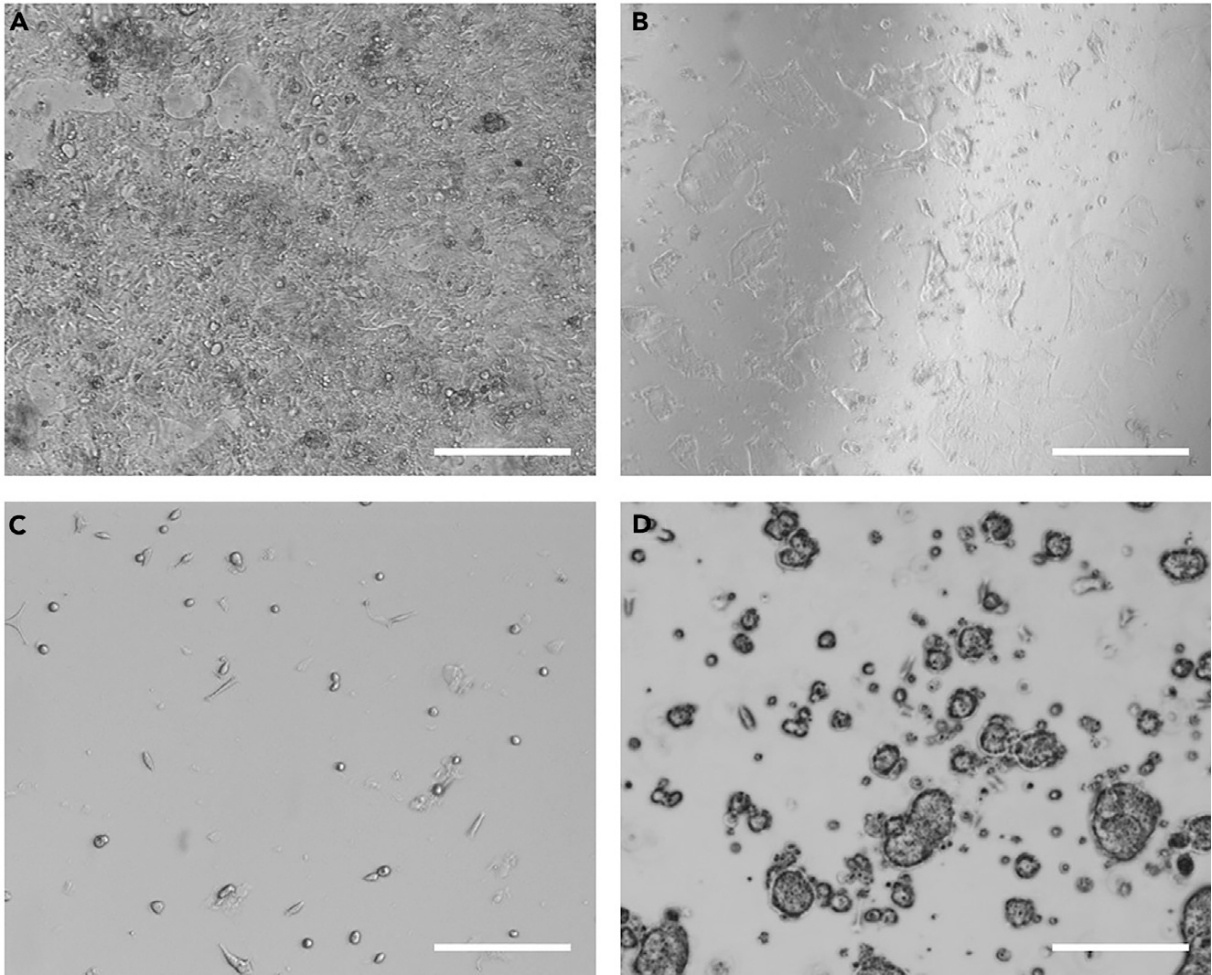
Sample FACS Layout Representative sort layout from 500k events, showing data from human ileal organoids expressing Venus under control of the proglucagon promoter (GLU-Venus) (Goldspink et al, 2020). Single cells were selected based on their side scatter (SSC) and forward scatter (FSC) (top row) and live single cells were further isolated from dead cells and debris based on DAPI and DRAQ5 fluorescence as indicated, before Venus-fluorescence positive (+ve) cells were distinguished from non-fluorescent negative (-ve) cells (bottom row).

### Extraction of FAC-Sorted Cells

**Timing: 21 h**

23. Thaw sorted cell samples on ice and then re-freeze on dry ice (or in a −70°C freezer) followed by defrosting on ice.
24. Add 900 μL of 80% ACN in water to each sample and vortex mix.
25. Centrifuge at 12,000 × *g* for 5 min at 4°C and transfer the aqueous phase (lower) to a clean LoBind Eppendorf.
26. Evaporate the supernatant in a rotary evaporator until dry (usually takes ~18 h; overnight evaporation).
27. Reconstitute in 500 μL 0.1% formic acid in water (v/v).
28. Transfer samples onto an Oasis HLB Prime μElution solid phase extraction (SPE) plate (Waters) and gently apply positive pressure on an SPE manifold.
29. Wash all samples with the following reagents, ensuring the SPE plate reservoirs are clear before each step.
  a. 200 μL 0.1% formic acid in water (v/v).
  b. 200 μL 5% methanol:1% acetic acid in water (v/v).
30. Elute the samples into a clean Quanrecovery plate with two 30 μL aliquots of 60% methanol:10% acetic acid in water (v/v).
31. Evaporate the supernatant under nitrogen at 40°C on a Biotage SPE Dry 96 system until dry.
32. Reconstitute into 75 μL of 10 mM DTT in 50 mM ammonium bicarbonate.
33. Incubate the samples for 1 h at 60°C.
34. Add 20 μL 100 mM iodoacetamide in 50 mM ammonium bicarbonate.
35. Incubate for 30 min at 18°C–25°C, in the dark.
36. Dilute the samples with 25 μL 1% formic acid in water (v/v).

### Generation of 2D Organoid Cultures

**Timing: 20 h**

This method describes the preparation of 2D organoid cultures for secretion experiments. The number of organoids required will depend on expression levels of the peptides of interest and differentiation conditions. To assess gut hormone secretion from intestinal organoid cultures (as described in Goldspink et al., 2020), we recommend that one confluent well of a 6-well plate is used to seed six wells of a 24-well plate.

37. Pre-coat plates with Matrigel.
  a. Thaw Matrigel aliquot on ice.
  b. Prepare an appropriate volume of 2% v/v Matrigel in ice-cold ADF.
  c. Add 300 μL of 2% Matrigel to the required number of wells of a 24-well plate.
  d. Incubate Matrigel-coated places at 37°C for approximately 1 h.
38. Meanwhile, recover organoids from matrix domes.
  a. Aspirate organoid culture media.
  b. Add 2 mL of ice-cold Advanced DMEM/F12 (ADF) per 6-well.
  c. Use a P1000 micropipette to scrape and collect organoids into a 15 mL centrifuge tube.
  d. Centrifuge at 500 × *g* for 5 min to pellet organoids then discard supernatant.
39. Resuspend organoid pellet in 4 mL of TrypLE Express per 6-well.
  a. Incubate in a water bath for 8–12 min at 37°C.
  b. Centrifuge at 400 × *g* for 5 min to pellet organoids then discard supernatant.
  c. Triturate 10–20 times with a P200 micropipette to mechanically disrupt the organoid pellet.

***Note:*** For optimal attachment, aim for small clusters of 10–20 cells (this can be visualized after plating or by directly observing cells in a 15 mL tube at any point under a light microscope).

40. Resuspend pellet in organoid culture media + 10 μM Y-27632 (250 μL/24-well, pre-warmed to 37°C) to obtain a cell suspension.
41. Aspirate Matrigel solution from pre-coated plate.
  a. Immediately add pre-warmed organoid culture media + 10 μM Y-27632 (250 μL/24-well) to each well.
42. Plate 250 μL of the cell suspension per well.

***Note:*** To ensure consistency between wells, mix the cell suspension by pipetting regularly during plating to ensure the suspension remains homogenous. After plating, gently shake plates (left to right and back to front) to ensure cells do not clump in the center of the well.

43. Incubate overnight (18–24 h) in an incubator (37°C, 5% CO_2_) to allow cell adhesion before stimulating secretion.

### Collection of 2D Secretion Supernatants

**Timing: 2 h**

This method describes the collection of supernatants from 2D organoid cultures stimulated under different conditions. This is performed in 24-well plates and it may be necessary to combine supernatants collected from multiple wells, depending on the peptides of interest and LC-MS sensitivity. To measure ileal gut hormone secretion (as described in Goldspink et al., 2020), we combine supernatants from three wells of a 24-well plate. Strong stimulants – such as forskolin/IBMX (each 10 μM) which elevate intracellular cAMP levels – can be used to assess secretion of peptides from a range of cell types. Alternatively, drugs which target specific receptors or molecular pathways can be tested to determine whether these stimulate or inhibit secretion of peptides of interest.

44. Following overnight incubation, observe cultures under a light microscope (Figure 2)

***Note:*** See Expected Outcomes and Troubleshooting 2

45. Add 0.001% fatty acid-free BSA and, if required, glucose (normally 10 mM) to an appropriate volume of saline buffer and pre-warm to 37°C (this solution is termed secretion buffer).
46. Wash cultures with pre-warmed secretion buffer.
  a. Aspirate overnight culture media.
  b. Add approximately 300 μL of warm secretion buffer to each well to wash cultures.
  c. Aspirate secretion buffer.
  d. Repeat steps b-c twice more then add a further 300 μL of warm secretion buffer to each well and incubate at 37°C for 30 min (meanwhile proceed to step 47).

***Note:*** These wash steps should not be too gentle as they aim to remove unattached cells or debris and wash off all culture media (which may interfere with LC-MS). The 30 min pre-incubation step allows acclimatization of cells to the low-nutrient secretion buffer.

47. Prepare stimulant solutions in secretion buffer (200 μL is required per well of a 24-well plate, and should be made up with 10% excess volume) and keep at 37°C.
48. After the 30 min pre-incubation, remove all the secretion buffer from wells (using a P1000 micropipette with the plate tilted to a 45° angle) and add 200 μL of stimulant solution to each well.

***Note:*** To avoid cells drying out, buffer should only be removed from 3–4 wells at a time

49. Incubate with stimulants at 37°C for 1 h.
  a. Meanwhile label and pre-chill (on ice) two LoBind 1.5 mL Eppendorf tubes per condition.
50. Collect supernatants into pre-chilled LoBind 1.5 mL Eppendorf tubes.

***Note:*** Supernatants from multiple wells can be combined at this step, if required for peptide detection by LC-MS. Steps 50–52 should be performed on ice using pre-chilled tubes to minimize peptide degradation

51. Centrifuge at 2,000 × *g* for 5 min at 4°C to pellet any debris or detached cells.
52. Transfer 180 μL (or greater volume if multiple wells were combined in step 50) of each supernatant to fresh LoBind tubes, taking care to avoid touching any pellet which forms.

**CRITICAL:** As intracellular levels of secreted peptides are normally higher than in supernatants, any detached cells will interfere with quantification

53. Immediately snap freeze on dry ice.

**Pause Point:** Samples can be frozen at −70°C until continuing with peptide extraction

### Extraction of Secretion Samples

**Timing: 1 h**

This method describes the extraction of peptides from organoid secretion supernatants.

54. Thaw secretion samples, if frozen, on ice.
55. To each sample, add 50 μL of 1% formic acid in water (v/v) and gently mix.

**CRITICAL:** Ensure steps 54 and 55 are performed on wet ice

***Note:*** If performing targeted quantitation of peptides prepare an internal standard solution in 1% formic acid in water (v/v) and add 50 μL instead of blank solvent

56. Transfer samples onto an Oasis HLB Prime μElution solid phase extraction (SPE) plate (Waters) and gently apply positive pressure on an SPE manifold.
57. Wash all samples with the following reagents, ensuring the SPE plate wells are clear before each step:
  a. 200 μL 0.1% formic acid in water (v/v)
  b. 200 μL 5% methanol:1% acetic acid in water (v/v)
58. Elute the samples into a clean Quanrecovery plate with two 30 μL aliquots of 60% methanol:10% acetic acid in water (v/v).
59. Dilute the samples with 75 μL 0.1% formic acid in water (v/v).

### Analysis of Peptides by Liquid Chromatography Mass Spectrometry

**Timing: 130 min per sample**

This step describes sample data acquisition of extracted samples by LC-MS analysis. Extracts are analyzed using a Thermo Fisher Scientific Ultimate 3000 nano LC system coupled to a Q Exactive Plus Orbitrap mass spectrometer according to the analytical parameters stated previously in LC-MS system set up.

60. Prior to injection onto the liquid chromatography (LC) system, centrifuge samples at 3,500 × *g* for 10 min.
61. Inject samples onto LC-MS system for analysis; recommended volumes of 30 μL for secretion supernatants and lysed organoids, and 40 μL for sorted cells.

***Note:*** See Troubleshooting 3 if chromatography is not as expected

### Quantification of Peptides in Raw LC-MS Data Files Using Qual Browser and Quan Browser Software

**Timing: up to 4 h depending on the number of LC-MS data files being searched**

62. Using PEAKS software (v8.5), open a representative LC-MS file in Qual Browser that will contain peptides of interest.
63. Extract the *m/z* ion specific for the peptide (as specified in the PEAKS software viewer) using the ranges function on the chromatogram pane (Figure 3B).

**Figure 3:**
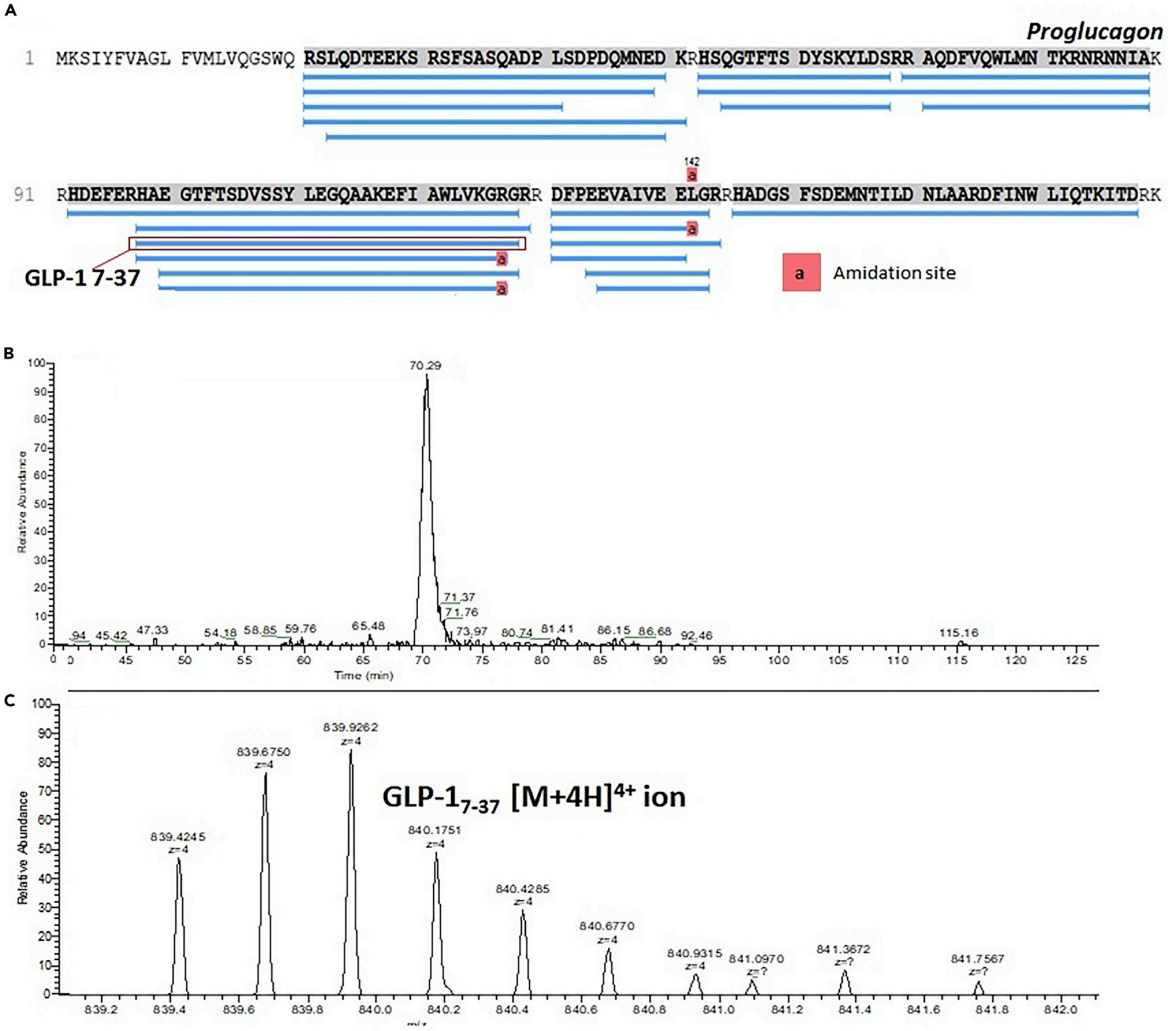
Peptides Matched against Proglucagon Sequence and Example GLP-1_7-37_ Chromatogram Results from a sample from Goldspink et al. (2020). (A) A PEAKS output showing the assignment of matched peptides against the proglucagon sequence from a lysed organoid sample. (B) Extracted ion chromatogram of *m/z* values relating to the [M + 4H]^4+^ charge state of the GLP-1 7-37 peptide generates a peak that can be integrated to semi-quantitatively monitor the peptide. (C) Ions relating to the [M + 4H]^4+^ charge state of the GLP-I 7-37 peptide.
64. Sum spectra under the peak at the expected retention time as specified in the PEAKS software (Figure 3C).
65. Note the *m/z* values of the peptide ^13^C isotopes for the peptide, usually the top 4 ions will suffice. Adding ions of low abundance can sometimes have little impact on signal, but can increase chromatogram noise.
66. Open Processing Setup program from the Xcalibur launch page.
67. Input peptide name, select MS full scan filter scan filter, *m/z* values retention time and chromatogram window settings as specified in Figure 4.

**Figure 4:**
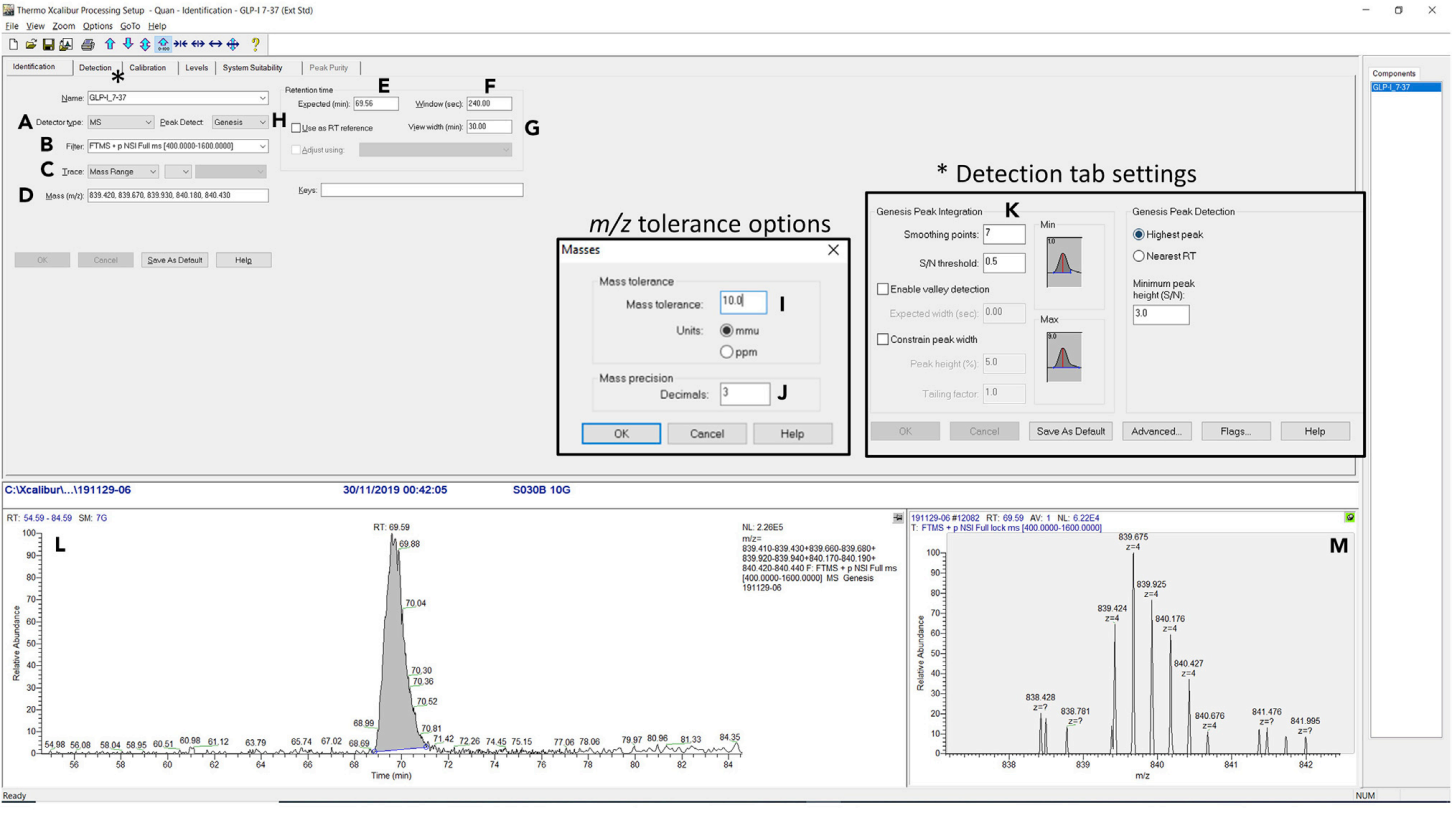
Procedure for Generating a Processing Method (A) Selection of LC-MS data function. (B) Selection of full scan data filter. (C) Enable mass range selection. (D) Input of specific *m/z* values for peptide of interest, a ± tolerance of 0.01 *m/z* is achieved by specifying 10 mmu in the “masses” function under the options tab (I), as well as the decimal place selection (J). (E) Expected peptide retention time. (F) Peptide selection time window tolerance. (G) Chromatogram view window range. (H) Integration algorithm selection. (K) Detection tab inset, showing selection of seven smoothing points (helps obtain more consistent peak integration). (L) Chromatogram showing peak integration using the selected *m/z* values and integration parameters. (M) Spectrum of peptide under integrated peak.
68. Open file list containing all LC-MS files and select batch reanalysis function to perform quantitative analysis of the data using the specified processing method.
69. Check integration of peptide peaks are correct, and if needed make changes to integration manually, or update the automatic integration parameters to reprocess all the integrations for a specific peptide (Figure 5).

**Figure 5:**
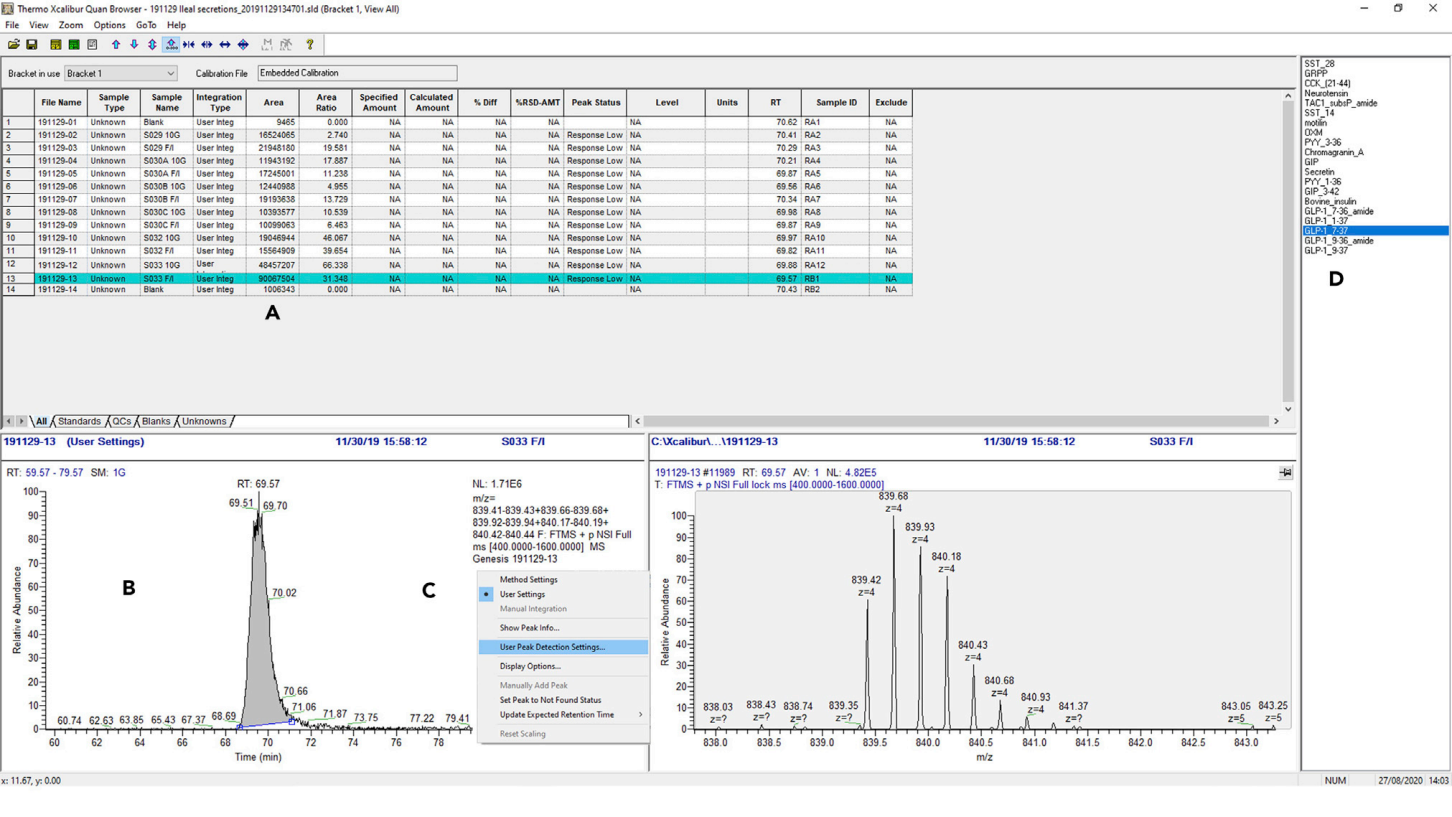
Procedure for Performing the Quantitative Analysis of Raw LC-MS Data Files (A) Peptide peak areas in analyzed samples. (B) Integration of peptide, which can be manually changed if needed. (C) User integration parameters can be modified and applied to all to improve automatic peptide integration. (D) Selection of specific peptide from list of target peptides.

## Expected Outcomes

Each sample injected onto the LC-MS system will generate a Total Ion Chromatogram (TIC) which is made up of all ionized species and can be filtered by mass ranges to identify the presence of specific peptide fragments. The data for each sample are processed using PEAKS software (v8.5) which matches experimentally acquired MS/MS spectra against proteins in the UniProt database (Figure 3A). Exporting data from the PEAKS software generates relative quantitative data at the protein level such that comparison between samples can be performed in other statistical software packages, such as RStudio.

See Figure 3 for an example of a peptide *m/z* isotope pattern and the identification of the specific retention time for peptide peak area integration purposes. This approach allows for the generation of semi-quantitative measurements of peptides – or fully quantitative if peptide standards are co-extracted and analyzed.

## Quantification and Statistical Analysis

To compare the levels of specific peptides, it is recommended that the raw data are interrogated manually. Each peptide generates a signature pattern in the MS data, and will acquire a varied number of charges to generate predictable mass to charge ratio (*m/z*) values. Close interrogation of each charge state will show that peptides will regularly have multiple ^13^C isotopes, therefore it is important to identify these and include them in the quantitative analysis to improve sensitivity.

Software packages that are supplied with the instrument, such as Qual Browser (Supplied in Xcalibur), can be used to interrogate the raw data to identify the specific *m/z* values for targeted peptide quantitation. Once the specific *m/z* patterns for each target peptide are identified, these can be used to quantify multiple peptides using other software packages, such as Quan Browser (Supplied in Xcalibur). Once the retention time of the peptide and the *m/z* values are identified, then the peak integration can be optimized for each peptide to ensure reproducibility in peak area assignment. Figures 4 and 5 shows an example of sample quantitation from Goldspink et al. (2020).

Peak area values can then be compared between samples in statistical software, such as RStudio.

## Limitations

Due to the chemical properties of peptides, not all peptides are extracted equally by this method and the extraction recovery and sensitivity of the LC-MS approach may vary for different peptides.

Nanoflow liquid chromatography runs take approximately 2 h to perform. This makes the method low throughput and therefore not suitable for large sample sets. The approach is also relatively expensive and requires the use of high cost and complex instrumentation.

The full scan peptidomic method described generates significant amounts of data (approximately 1 GB per 2 h analysis), which can be time consuming to process. Additionally, this type of measurement will identify all ionized species in the samples which may include unwanted contaminants, such as BSA, media components, and peptides from typical cell turnover processes. These may compete for MS/MS fragmentation selection with peptides of interest, which will reduce the depth of peptide identification in samples. If data are only required on known peptides, a more targeted analysis using a triple quadrupole MS system can be used instead of a high-resolution mass spectrometer. These systems are specifically designed to be used for quantitative peptidomics analysis and are usually employed in a higher throughput manner compared to traditional discovery peptidomics methods.

## Troubleshooting

### Problem 1

Poor LC-MS chromatographical peak shape and sensitivity (step 61).

### Potential Solution

Over time, the efficiency of LC-MS consumables (columns and traps) can decline, this leads to poor chromatographic separation, shifts in retention time, and broadening of peptides which results in a reduction in the sensitivity of the LC-MS.

Regular checks on the suitability of the LC-MS system should be performed by analyzing a standard peptide solution and monitoring the peak retention times and signal of key peptides. Trap column cartridges can be replaced quickly, while new analytical columns require more effort to get running again.

Over time the MS instrument might accumulate dirt on the orifice, reducing sensitivity which will require the transfer tube to be removed and cleaned.

### Problem 2

Poor formation of 2D cultures (step 43).

### Potential Solution

When plating 2D cultures, organoids should be broken up into small clusters of 10–20 cells. If organoids are not sufficiently broken up, then 3D structures may reform during overnight incubation. By contrast, if organoids are broken down too harshly, they form sparse or single-cell cultures. Ensure Y-27632 is added to culture media during plating to limit anoikis-induced cell death. Plating density must be optimized based on organoid size and number of organoids per well. Some differentiation-promoting compounds (such as Notch inhibitors) may also interfere with cellular attachment. Cultures should be plated on a thin coat of Matrigel (2%) to improve attachment. Increasing the Matrigel pre-coating period from 1 h to up to 8 h may improve poor cellular attachment. See Figure 2for a comparison of successful and unsuccessful organoid cultures.

### Problem 3

Expected peptides not detected by LC-MS (step 61).

### Potential Solution

This could be caused by poorly differentiated organoids lacking mature cell types. Organoid culture protocols should be optimized prior to peptidomic analyses using techniques such as qPCR, immunostaining, and the presence of fluorescent reporters. Methods used for culture of human small intestinal organoids are described in Goldspink et al, 2020.

LC-MS analyses are generally less sensitive than antibody-based methods and therefore a large number of organoids are normally required for sample extraction, depending on the expression levels of the peptides of interest. We recommend analyzing organoid lysates by LC-MS/MS to make sure they are producing the peptide of interest before attempting experiments using sorted cells or secretion supernatants. Detection of gut hormone peptides can be improved by using culture protocols which selectively drive the enteroendocrine lineage (Beumer et al., 2020, Goldspink et al., 2020).

In some instances, peptides may not be found during database searches which can be caused by peptide degradation. The presence of breakdown products can be used to confirm if this has occurred during sample extraction. The use of short incubation periods and performing sample collection on ice is more effective at preventing over-fragmentation than the addition of protease inhibiting peptides. The addition of formic acid immediately prior to extraction after thawing will help reduce proteolytic activity, while the addition of 6 M GuHCl to the homogenate analysis will also prevent degradation during extraction.

## Resource Availability

### Lead Contact

Further information and requests for resources and reagents should be directed to and will be fulfilled by the Lead Contact, Richard Kay (rgk27@medschl.cam.ac.uk).

### Materials Availability

No new materials were generated in this study.

### Data and Code Availability

The sorted cell peptidomics data have been deposited to the ProteomeXchange Consortium via the PRIDE partner repository (PXD017825).

## References

[bib1] Beumer J., Puschhof J., Bauza-Martinez J., Martinez-Silgado A., Elmentaite R., James K.R., Ross A., Hendriks D., Artegiani B., Busslinger G.A. (2020). High-resolution mRNA and secretome atlas of human enteroendocrine cells. Cell.

[bib2] Fujii M., Matano M., Nanki K., Sato T. (2015). Efficient genetic engineering of human intestinal organoids using electroporation. Nat. Protoc..

[bib3] Goldspink D.A., Lu V.B., Miedzybrodzka E.L., Smith C.A., Foreman R.E., Billing L.J., Kay R.G., Reimann F., Gribble F.M. (2020). Labeling and characterization of human GLP-1-secreting L-cells in primary ileal organoid culture. Cell Rep..

